# Differential Expression of MicroRNA (MiR-27, MiR-145) among Dental Pulp Stem Cells (DPSCs) Following Neurogenic Differentiation Stimuli

**DOI:** 10.3390/biomedicines11113003

**Published:** 2023-11-09

**Authors:** Charlton Bassett, Hunter Triplett, Keegan Lott, Katherine M. Howard, Karl Kingsley

**Affiliations:** 1 School of Medicine, University of Nevada, Las Vegas 1700 West Charleston Boulevard, Las Vegas, NV 89106, USA; bassec1@unlv.nevada.edu (C.B.); triplh1@unlv.nevada.edu (H.T.); lottk1@unlv.nevada.edu (K.L.); 2 School of Dental Medicine, University of Nevada, Las Vegas 1001 Shadow Lane, Las Vegas, NV 89106, USA; katherine.howard@unlv.edu

**Keywords:** mesenchymal stem cell (MSC), dental pulp stem cell (DPSC), microRNA, stem cell differentiation

## Abstract

This study sought to evaluate the expression of previously identified microRNAs known to regulate neuronal differentiation in mesenchymal stem cells (MSCs), including miR-27, miR-125, miR-128, miR-135, miR-140, miR-145, miR-218 and miR-410, among dental pulp stem cells (DPSCs) under conditions demonstrated to induce neuronal differentiation. Using an approved protocol, *n* = 12 DPSCs were identified from an existing biorepository and treated with basic fibroblast growth factor (bFGF) and epidermal growth factor (EGF), which were previously demonstrated to induce neural differentiation markers including Sox1, Pax6 and NFM among these DPSCs. This study revealed that some microRNAs involved in the neuronal differentiation of MSCs were also differentially expressed among the DPSCs, including miR-27 and miR-145. In addition, this study also revealed that administration of bFGF and EGF was sufficient to modulate miR-27 and miR-145 expression in all of the stimulus-responsive DPSCs but not among all of the non-responsive DPSCs—suggesting that further investigation of the downstream targets of these microRNAs may be needed to fully evaluate and understand these observations.

## 1. Introduction

Many research groups have recently focused their attention and efforts into bioengineering and tissue regeneration using mesenchymal stem cells (MSC) derived from dental pulp, known as dental pulp stem cells (DPSCs) [1,2]. Many types of oral and dental stem cells (DSC) have been identified, including location-specific periodontal ligament stem cells (PDLSC) and stem cells from the apical papilla (SCAP), as well as stem cells from exfoliated deciduous teeth (SHED) and DPSCs derived from vital, intact permanent teeth, which function in vivo to regenerate tissues and structures including the dentin-pulp complex [1,2]. Although all are derived from neural crest cells, the proliferative and differentiation potential may be different among these various types of DSC and DPSCs [1,2]. Due to these differences, many of these research and evaluation efforts have been highly concentrated on evaluating the potential to repair and regenerate specific dental or oral tissues and structures, such as PDLSCs and SCAPs to restore alveolar tissues and supporting bone structures or SHEDs and DPSCs to initiate tooth regeneration and other types of oral tissue remodeling [3,4]. These studies have evaluated and subsequently demonstrated highly successful methods and models for DPSCs and DSCs to regenerate and biofabricate some of these oral tissues and tooth structures in vitro and ex vivo [5,6].

However, a growing number of research groups have demonstrated that DPSCs are capable of regenerating and repairing other types of tissues outside of the oral cavity [7,8]. For example, significant progress has been made toward DPSC models used in osteogenic and bone remodeling [9,10]. Similarly, other research has demonstrated that DPSCs may be useful in the repair or remodeling of vascular structures or perivascular tissues through complex interactions observed within the process of angiogenesis [11,12]. Most recently, DPSCs have demonstrated the potential to be useful in the possible treatment and regeneration of neural disorders [13,14].

Due to the increasing prevalence of neurologic disorders associated with chronic conditions and associated diseases of aging, such as Parkinson’s and Alzheimer’s disease, interest has been developing to find clinical and translational uses for MSC and DPSC neuron-specific treatment, repair, and regeneration [15,16]. Although the standardization of procedures and protocols has not yet been established, the basic principles and guidelines are now available to direct the development and differentiation of DPSCs and MSCs into neuronal precursors [17,18]. Due to the lack of standardization in protocols and the variety of induction mechanisms available, more efforts are being made to understand the common underlying signaling pathways and processes responsible for neuronal regeneration and induction [19,20,21].

New evidence has suggested that many of the differing methods and mechanisms used to induce differentiation in DPSCs may actually converge in common or shared regulatory mechanisms, such as the expression and modulation of non-coding microRNAs [22,23,24,25]. It has been established that these short, non-coding microRNAs may function to mediate and modulate the gene expression in many types of cells and tissues, and many microRNAs are now known to direct and regulate the osteogenic differentiation of DPSCs through both positive (induction) and negative (inhibitory) regulatory feedback mechanisms, such as miR-24, miR-31, miR-140, miR-143, miR-153, and miR-188 [26,27,28,29,30,31]. However, the mechanisms and processes of microRNA expression that regulate neuronal differentiation in DPSC are virtually unknown—although miR-27, miR-125, miR-128, miR-135, miR-140, miR-145, miR-218, and miR-410 have been identified as potential regulators of MSC neuronal differentiation [32,33,34,35,36,37,38].

More specifically, miR-125 and miR-128 have been demonstrated to potentiate the differentiation and specification of neural progenitors among embryonic and amniotic stem cells [32,33]. In contrast, miR-135, miR-140, and miR-145 appear to primarily function in differentiated and functionally distinct neuronal cells—although some evidence may suggest their involvement in adult neurogenesis [34,35,36]. Finally, other microRNAs have been specifically identified for their potential to modulate neurogenesis within MSC in vivo or ex vivo, including miR-218 and miR-410 [37,38].

Based on the paucity of evidence, the primary objective of this study was to evaluate the expression of previously identified microRNAs known to regulate neuronal differentiation in MSCs, including miR-27, miR-125, miR-128, miR-135, miR-140, miR-145, miR-218, and miR-410, among DPSCs under conditions demonstrated to induce neuronal differentiation [39].

## 2. Materials and Methods

### 2.1. Project Approval

This retrospective analysis of DPSCs used an existing biorepository from the University of Nevada, Las Vegas (UNLV)—School of Dental Medicine (SDM). The project protocol was reviewed by the Office for the Protection of Research Subjects (OPRS) and the Institutional Review Board (IRB) at UNLV. Approval for “Retrospective analysis of dental pulp stem cells (DPSC) from the University of Nevada, Las Vegas (UNLV) School of Dental Medicine (SDM) pediatric and clinical population” was designated on 21 February 2021 under OPRS Protocol #171612-1.

### 2.2. Original Study Approval and Sample Collection

The samples contained within the biorepository were originally collected under an approved study from the UNLV-OPRS and IRB under protocol OPRS#0907-3148 “Isolation of Non-Embryonic Stem Cells from Dental Pulp,” which was approved on 5 February 2010. All the samples were collected from UNLV-SDM clinic patients, who were unpaid and voluntary participants. The original protocol inclusion criteria were listed as only allowing UNLV-SDM patients of record to participate who had been previously scheduled to have an extraction of one or more vital, permanent, undamaged teeth as a pre-scheduled part of an active and continuing orthodontic therapy treatment plan to relieve overcrowding. All participants over 18 years of age were required to provide informed consent. Any patient under 18 years of age was required to provide pediatric assent in addition to the informed consent of the parent or guardian at the time of treatment. The exclusion criteria were listed as any person who was not a UNLV-SDM patient of record, any patient who declined to participate, and any patient who was scheduled for tooth extraction due to an alternative criterion, such as decay or disease (e.g., fracture, decay, caries, periodontal disease).

In brief, each participant included in the studies provided informed consent and participated voluntarily. All participants had to meet the inclusion criteria, which included being registered patients at the UNLV School of Dental Medicine (SDM) clinic and having an appointment scheduled for the extraction of permanent, premolar, or third molar teeth as a standard component of an existing orthodontic therapy treatment plan. Exclusion criteria consisted of individuals who were not registered patients at UNLV-SDM, declined to participate, or required tooth extractions for alternative reasons, such as severe dental decay, periodontal disease, or fractures.

### 2.3. Original Sample Processing and Storage

For the transfer to a biomedical laboratory, the dental pulp was removed from each extracted tooth and placed into microcentrifuge tubes consisting of a sterile phosphate-buffered saline (PBS) to facilitate the transfer. All the DPSCs were placed into Gibco Advanced Roswell Park Memorial Institute (RPMI) media with 10% bovine growth serum (BGS) and 1% penicillin-streptomycin, all from Thermo Fisher Scientific (Waltham, MA, USA), and allowed to proliferate using the previously described direct outgrowth method, which involves the sectioning and placement of dental pulp into culture without enzymatic digestion or other biochemical modifications [40]. Each DPSC isolate was subsequently allowed to proliferate in culture for a minimum of ten (*n* = 10) passages before freezing, allowing for the self-selection of stem cells in culture with extended proliferative capacity. The presence of mesenchymal stem cell (MSC) biomarkers CD90 and CD105 were verified among all DPSC isolates, as was the lack of expression for the negative control biomarker CD45, as outlined by the specifications of the International Society for Cellular Therapy (ISCT), as described previously [39,40]. A standardized cell freezing protocol was then used for cryopreservation, with the cell culture media containing 10% dimethyl sulfoxide (DMSO) and the addition of Fetal Bovine Serum (FBS), as previously described [40].

### 2.4. Current Study Protocol

DPSC isolates from the existing biorepository were identified for inclusion in this study (*n* = 12). All the DPSC isolates were thawed slowly over 60 min on ice and subsequently centrifuged for five minutes at 2100× *g* or relative centrifugal force (RCF) for removal of the DMSO-containing freezing medium prior to placing the cells into the culture. Recent improvements to the cell culture from this group have established a differentiated culture protocol using DPSC isolate-specific optimized media for culturing, which may involve Roswell Park Memorial Institute (RPMI), Dulbecco’s Modified Eagle’s Medium (DMEM), alpha-Minimum Essential Media (MEM), or DMEM:Nutrient Mixture F12 supplemented with 10% BGS and 1% penicillin-streptomycin, as previously described [40,41].

### 2.5. Growth Factor Administration

To induce MSC or DPSC differentiation in a neural lineage, epidermal growth factor (EGF) and basic fibroblast growth factor (bFGF) were used, as previously described [40,42,43]. Experimental growth factors, including bFGF (#13256029) and EGF (#PHG0311), were obtained from Gibco/Thermo Fisher Scientific (Waltham, MA, USA). Administration of growth factors was standardized at 20 ng/mL, which was consistent with other studies of neural differentiation [40,42,43]. Non-treated controls used 1× phosphate-buffered saline (PBS) instead of EGF and bFGF in the cell culture media protocols.

### 2.6. Viability and Proliferation Assays

The viability of each DPSC isolate was performed under experimental and control conditions with 0.4% Trypan Blue Solution obtained from Fisher Scientific (Fair Lawn, NJ, USA), as previously described [39,40]. In brief, equal volumes of the DPSC cell culture media were mixed with Trypan Blue and analyzed using a BioRad TC20 automated cell counter (Hercules, CA, USA). Measurements of the percentage of live cells were evaluated throughout the cell culture and during all experimental assays.

The proliferation of each DPSC isolate was assessed under both experimental and control conditions using standardized 96-well growth assays. In brief, equal numbers of DPSC cells (1.2 × 10^5^ cells/mL) were plated in Corning Costar 96-well flat bottom assay plates from Fisher Scientific (Fair Lawn, NJ, USA) with and without growth factors for three days (72 h). Cells were then fixed using 10% buffered formalin for a minimum of 24 h. The formalin was subsequently removed by aspiration and the cells were processed using 1% Gentian Violet stain (Fisher Scientific; Fair Lawn, NJ, USA). The Gentian Violet was removed and the plates were washed using 1× phosphate-buffered saline (PBS) prior to evaluation. The analysis was completed with an ELx808 96-well plate reader from Eppendorf (Hamburg, Germany) and the specific wavelength filter at 630 nm. Absorbance data were saved and exported for analysis.

### 2.7. RNA Isolation

To determine if the addition of the growth factors (EGF, bFGF, combination) induced any changes to the microRNA expression, the total RNA was extracted from each of the DPSC isolates under control (non-treatment) and experimental (treated) conditions, as previously described [39,40,41]. In brief, TRIzol RNA isolation reagent (500 µL) from Fisher Scientific (Fair Lawn, NJ, USA) was mixed with an equal volume of DPSC cell suspension (500 µL) and triturated before adding chloroform (200 µL), also from Fisher Scientific (Fair Lawn, NJ, USA). Each sample was incubated on ice for 15 min and then centrifuged in a refrigerated 5424 Microcentrifuge from Eppendorf (Hamburg, Germany) at 12,000× *g* or RCF at 4 °C. The RNA from the aqueous or upper phase (300 µL) was moved into a new microcentrifuge tube and combined with isopropanol (300 µL) from Fisher Scientific (Fair Lawn, NJ, USA) to facilitate the precipitation of nucleic acids. All samples were then centrifuged at 12,000× *g* or RCF at 4 °C for an additional ten minutes and the supernatant was removed by aspiration. The RNA-containing pellet was washed with ethanol from Fisher Scientific (Fair Lawn, NJ, USA) and centrifuged once again. Following removal of ethanol, resuspension of the pellet was accomplished using nuclease-free water (100 µL). Quantification of RNA quantity and quality was performed using the NanoDrop 2000 Spectrophotometer (Fisher Scientific; Fair Lawn, NJ, USA) using absorbance readings at A260 and A280 nm prior to short-term storage at −20 °C or long-term storage at −80 °C.

### 2.8. cDNA Synthesis

Conversion of the RNA into cDNA was facilitated using the ABgene Reverse-iT One-Step RT-PCR kit from Fisher Scientific (Fair Lawn, NJ, USA). In brief, RNA from each DPSC isolate (control, treatment) was added to reactions prepared according to the manufacturer recommendations, as previously described [39,40,41]. RT-PCR Master mix (Reddy Mix from ABgene kit) was prepared with DPSC total RNA (1.0 µg). To each reaction, random primers (forward and reverse) obtained from Invitrogen (Waltham, MA, USA) were added with RTase blend and the addition of nuclease-free water to a standardized volume of 25 µL. All reactions were processed using a Mastercycler gradient thermal cycler from Eppendorf (Hamburg, Germany) and the recommended settings of 47 °C for 30 min for reverse transcription, with final extension for five minutes at 72 °C. Quantitative and qualitative assessment of the resulting cDNAwas performed using a NanoDrop 2000 Spectrophotometer (Fisher Scientific; Fair Lawn, NJ, USA) using absorbance readings at A260 and A280 nm prior to short-term storage at −20 °C or long-term storage at −80 °C.

### 2.9. microRNA Processing

Due to the potential for low expression among the microRNA targets, the conversion and amplification of microRNA from the DPSC isolates was processed using the TaqMan Advanced miRNA Assay conversion kit (ThermoFisher; Fair Lawn, NJ, USA). Briefly, the first step of the protocol was the Poly-A tailing reaction that included Poly(A) buffer, ATP, Poly(A) enzyme, and RNase-free water combined into the completed Poly(A) reaction mix according to the manufacturer-recommended protocol. This Poly(A) reaction mix was added to the wells of a 96-well qPCR reaction plate and then incubated in a thermal cycler for 45 min at 37 °C, and then for an additional 10 min at 65 °C.

The second step of the protocol was the adaptor ligation reaction, involving DNA Ligase buffer, 50% PEG 8000, The ligation adapter, RNA ligase, and RNase-free water combined into a Ligation Reaction Mix following the manufacturer-recommended protocol. The Ligation Reaction Mix was subsequently added to the Poly(A) tailing reaction product in the reaction plate and then incubated in the thermal cycler for 60 min at 16 °C.

The third step of the protocol was the reverse transcription (RT) reaction, which included RT buffer, dNTP mix, universal RT primer, RT enzyme mix, and RNase-free water, which were combined into an RT Reaction Mix according to the manufacturer protocol and then added to the adaptor ligation reaction product in the reaction plate. This plate was incubated for 15 min at 42 °C followed by an additional five minutes at 85 °C.

The fourth and final step of the protocol was to use the cDNA derived from the reverse transcription reaction and further amplify these by using the TaqMan miR-Amp Reaction Mix (Applied Biosystems; Waltham, MA, USA). In brief, the manufacturer-recommended protocol was followed, which included the use of the miR-Amp Master Mix, 20X Primer Mix and nuclease and RNase-free water to create the reaction mix. The Mastercycler gradient thermal cycler (Eppendorf; Hamburg, Germany) was subsequently used using the recommended settings, which included an initial enzyme activation cycle for five minutes at 95 °C, followed by denaturation involving 14 cycles at 95 °C, followed by extension for 30 s performed at 60 °C, with the stop reaction completed for ten minutes at 99 °C.

### 2.10. qPCR Screening

The analysis of the DPSC microRNA expression was facilitated using the SYBR Green Real-Time PCR Master Mix from ThermoFisher Scientific (Fair Lawn, NJ, USA), following the manufacturer-recommended protocol as previously described [39,40,41]. In brief, qPCR reactions utilized the SYBR Green PCR Master Mix (12.5 µL), cDNA from each DPSC isolate (control, experimental), and validated primers (forward and reverse) that were specific for the specific target (Table 1). All reactions were processed using the QuantStudio 5 Real-Time PCR system (Fisher Scientific; Fair Lawn, NJ, USA) and the recommended protocol, including initial enzyme activation for ten minutes at 95 °C, denaturation for fifteen seconds at 95 °C, followed by forty cycles of annealing and denaturation using validated primer pair-specific temperatures listed below.

Primers included the positive qPCR internal control genes, glyceraldehyde 3-phosphate dehydrogenase or GAPDH, and beta actin [39,40]. MSC biomarker primers included NANOG, Nestin, Oct4, and Sox2 [39,40]. Neuronal biomarkers included beta III tubulin, neurofilament M or NFM, Pax6, Sox1, and Vim, as described previously [39]. Validated microRNA primers associated with the neuronal differentiation included miR-27, miR-125, miR-128, miR-135, miR-140, miR-145, miR-218, and miR-410—as well as the positive control microRNA, miR-16 [32,33,34,35,36,37,38]. All the primers were obtained from Thermo Fisher Scientific (Fair Lawn, NJ, USA) and were synthesized by SeqWright (Houston, TX, USA).

### 2.11. Statistical Analysis

Quantitative data from the proliferation and viability assays, as well as the quantitative data from the RNA and cDNA experiments, were analyzed using Microsoft Excel, Version 2019 (Redmond, WA, USA). The differences between the averages of the DPSC isolates (rDT, iDT, sDT) were evaluated using two-tailed Student’s *t*-tests, which are appropriate for parametric, continuous data analysis. The statistical significance levels were set at alpha (α) = 0.05.

## 3. Results

All the DPSC isolates selected for inclusion in this study (*n* = 12) were placed into the cell culture to confirm the baseline growth rates (Table 2). These data revealed that five DPSC isolates showed rapid doubling times of approximately two days, including dpsc-7089 (1.9 days), dpsc-3882 (1.8 days), dpsc-5653 (1.9 days), dpsc-9765 (2.1 days), and dpsc-3924 (2.2 days). Three DPSC isolates were found to exhibit intermediate doubling times of approximately five days, which included dpsc-8604 (5.1 days), dpsc-8124 (5.4 days) and dpsc-9894 (5.2 days). The remaining four DPSC isolates exhibited slow doubling times of ten to twelve days, such as dpsc-11418 (10.2 days), dpsc-17322 (10.6 days), dpsc-11836 (12.1 days), and dpsc-11750 (11.9 days). Viability was also assessed and varied between 50 and 77%, with the average viability of the rDT (66.4%) not differing significantly from the iDT (56.3%, *p* = 0.166) or the sDT (59.5%, *p* = 0.109) DPSC isolates.

**Figure 1:**
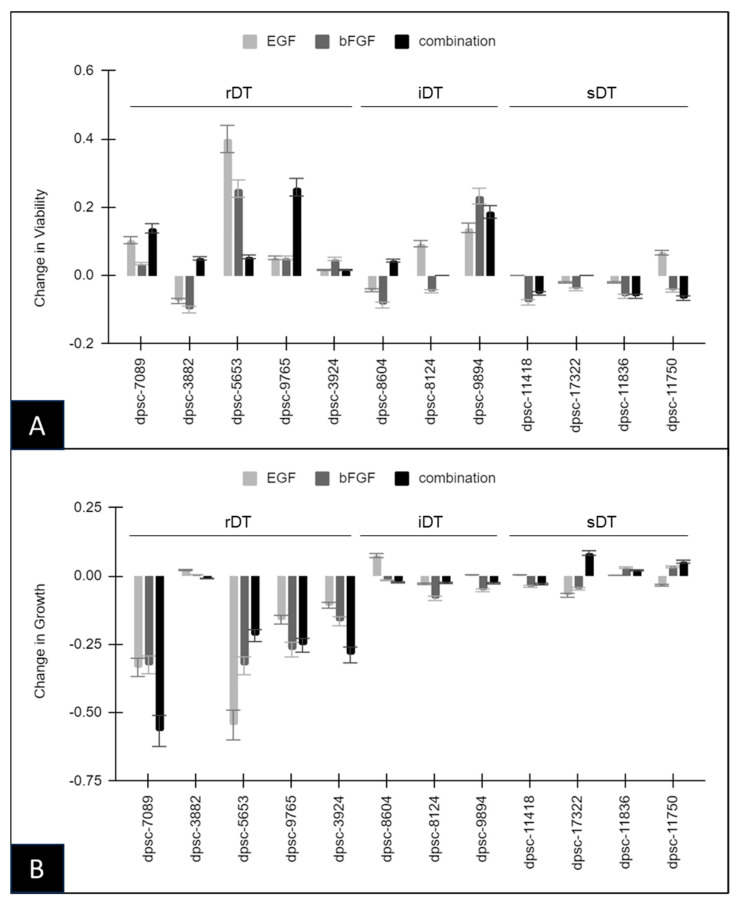
Analysis of the DPSC growth and viability. (**A**) Assessment of the DPSC viability under growth factor administration with EGF, bFGF, or combination treatment induced significant changes among most of the rapid dividing time (rDT) isolates including dpsc-7089, dpsc-3882, dpsc-5653, and dpsc-9765. Some iDT isolates were also responsive, including dpsc-8604 and dpsc-9894. (**B**) Assessment of the DPSC proliferation under the growth factor administration. Treatment of the DPSC isolates with EGF, bFGF, or combination treatment induced significant changes among most of the rapid dividing time (rDT) isolates including dpsc-7089, dpsc-5653, dpsc-9765, and dpsc-3924. Some iDT isolates were also responsive, including dpsc-8604 and dpsc-8124.

To assess the previously observed effects of th bFGF and EGF administration, proliferation and viability assays were performed on all the DPSC isolates with and without treatment, *n* = 12 (Figure 1). These data demonstrated that most rDT DPSC isolates were responsive to EGF, bFGF, or both in combination. More specifically, the viability was altered significantly compared with the baseline (control) viability under the EGF administration with the rDT isolates dpsc-7089 (10.3%), dpsc-3882 (−7.5%) dpsc-5653 (40.1%), dpsc-9765 (5.2%), as well as the iDT isolates dpsc-8124 (9.3%) and dpsc-9894 (12.9%) (Figure 1A). Changes in the viability under bFGF administration were observed with the rDT isolates dpsc-3882 (−10%), dpsc-5653 (25.5%), and dpsc-9765 (5.1%), with some alterations also observed among the iDT isolates dpsc-8604 (−8.6%) and dpsc-9894 (23.2%). Finally, the combination of the EGF and bFGF treatment induced significant changes in the viability among most of the rDT isolates, such as dpsc-7089 (12.8%), dpsc-3882 (5.0%), dpsc-5653 (5.5%), dpsc-9765 (25.8%), as well as the iDT isolate dpsc-9894 (18.6%).

To further evaluate the previously observed effects of the EGF and bFGF administration, both alone and in combination, the proliferation assays were performed on all the DPSC isolates with and without treatment, *n* = 12 (Figure 1B). According to the data, the majority of the rapidly dividing or rDT DPSC isolates were also shown to be responsive to EGF, bFGF, or both in combination. More specifically, the proliferation was altered and reduced compared with the baseline (control) growth under the EGF administration with the rDT isolates dpsc-7089 (−33.4%), dpsc-5653 (−54.5%), dpsc-9765 (−15.9%), and dpsc-3924 (−10.6%) as well as the iDT isolate dpsc-8604 (7.4%). Similarly, changes in the proliferation under bFGF administration were observed with the rDT isolates dpsc-7089 (−32.5%), dpsc-5653 (−32.9%), and dpsc-9765 (−26.9%), and dpsc-3924 (−16.5%) with some alterations also observed among the iDT isolate dpsc-8124 (−8.1%). Finally, the combination of the EGF and bFGF treatment induced significant changes in the proliferation among most of the rDT isolates, such as dpsc-7089 (−56.7%), dpsc-5653 (−21.8%), dpsc-9765 (−25.3%), and dpsc-3924 (−28.9%).

To evaluate any changes in the microRNA expression, the RNA was extracted from all the DPSC isolates (Table 3). These data demonstrated that the RNA concentrations ranged between 302 ng/µL and 577 ng/µL, with the yield between the rDT (average 406.6 ng/µL), iDT (average 442 ng/µL), and the sDT (average 472.8 ng/µL) DPSCs not significantly different, *p* = 0.329. In addition, the average RNA purity as measured by the A260:A280 ratio was also similar between the rDT (average 1.78), iDT (average 1.78) and sDT (average 1.82) DPSC isolates, *p* = 0.504.

Analysis of the cDNA synthesized from the RNA templates ranged between 1480 and 1610 ng/µL, with no significant differences between the averages of the rDT (1548.2 ng/µL), iDT (1567.0 ng/µL), and sDT (1549.5 ng/µL), *p* = 0.975. Furthermore, the average cDNA purity, measured by the A260:A180 ratio, was also not significantly different between the rDT (average 1.89), iDT (average 1.83), and sDT (average 1.87) DPSC isolates, *p* = 0.527.

**Figure 2:**
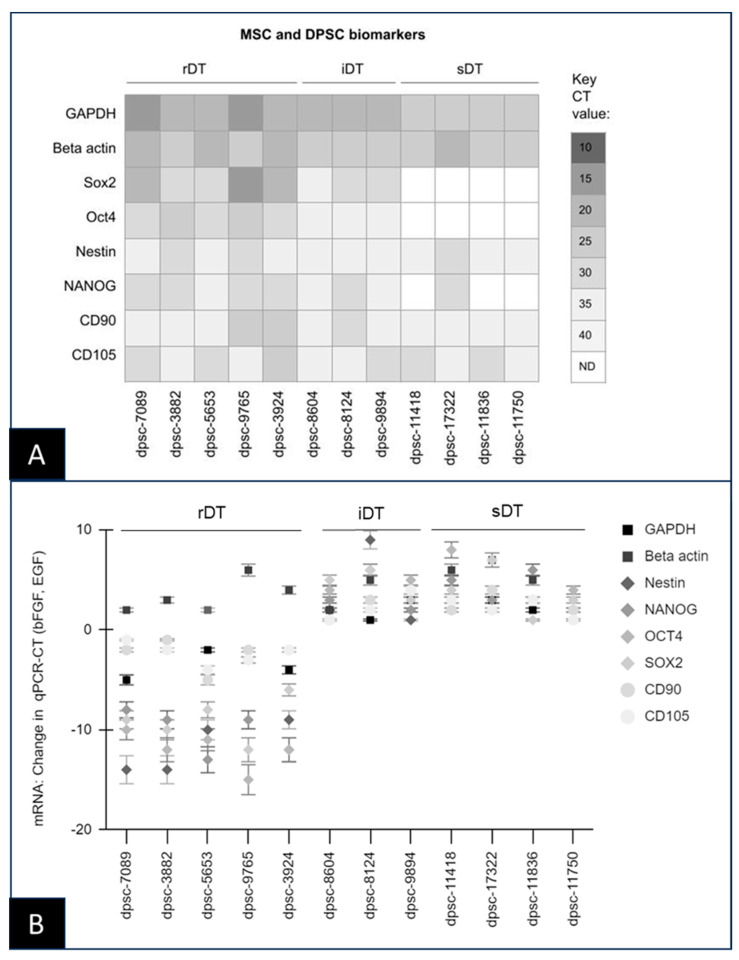
qPCR screening of the DPSC mRNA expression. (**A**) All of the rDT, iDT, and sDT DPSC isolates expressed the internal controls GAPDH and beta actin, as well as the ISCT positive control markers CD90 and CD105. Expression of the MSC biomarkers Sox2, Oct4, Nestin, and NANOG were observed among all of the rDT and iDT isolates, with only NANOG observed among the sDT isolate dpsc-17322. (**B**) Administration of bFGF and EGF altered expression of the MSC and ISCT biomarkers among the rDT but not iDT or sDT DPSC isolates with no observed changes in expression of the internal controls.

Screening of the RNA extracted from the DPSC isolates revealed the expression of several important MSC biomarkers and reference genes (Figure 2). More specifically, the internal control reference genes, including the positive control metabolic standard glyceraldehyde 3-phosphate dehydrogenase (GAPDH) as well as the structural marker beta actin, were highly expressed among all the DPSC isolates (Figure 2A). In addition, the MSC biomarker Nestin was expressed in all the DPSC, although differential expression of additional MSC biomarkers Sox2, Oct4, and NANOG was observed. The biomarkers Sox2, Oct4, and NANOG were expressed among all the rDT and iDT DPSC isolates, although no expression was found among the sDT DPSC isolates with the noted exception of NANOG and dpsc-17322.

However, administration of the bFGF and EGF induced differential changes in mRNA expression among some of the DPSC isolates (Figure 2B). More specifically, the addition of these growth factors altered the expression of the MSC biomarkers Sox2, Oct, Nestin, and NANOG among the rDT DPSC isolates (dpsc-7089, dpsc-3882, dpsc-5653, dpsc-9765, dpsc-3924), but not among the iDT or sDT DPSC isolates. Similar changes were observed with the DPSC biomarkers CD90 and CD105 among the rDT DPSC isolates, but not among the iDT or sDT DPSC isolates.

**Figure 3:**
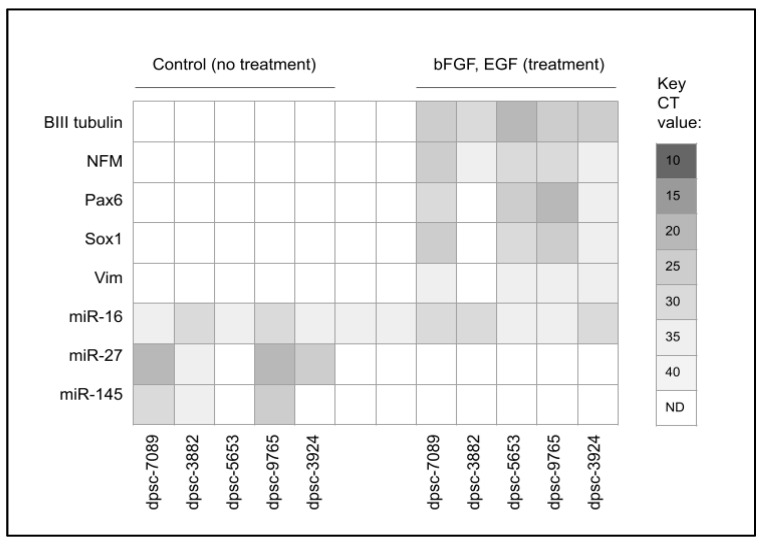
qPCR screening of the DPSC neuronal biomarker and microRNA expression. Expression of the neuronal biomarkers (BIII tubulin, NFM) was observed among the post-treatment DPSC isolates, with differential expression of Pax6, Sox1 and Vim observed. Expression of miR-27 and miR-145 was observed among some pre-treatment DPSC isolates, but not among any of the post-treatment samples. No expression of miR-125, miR-128, miR-135, miR-140, miR-218, or miR-410 was observed in either the pre- or post-treatment groups.

To determine the effect of bFGF and EGF treatment on the differentiation of the DPSC isolates, biomarkers for neuronal differentiation were evaluated among the rDT growth factor-responsive DPSC isolates (Figure 3). These data demonstrated that none of the biomarkers evaluated (beta tubulin, NFM, Pax6, Sox1, or Vim) were expressed in the pre-treatment DPSC isolates. However, the rDT bFGF and EGF-responsive DPSC isolates expressed beta III tubulin and neurofilament (NFM). In addition, differential results were observed with Pax, Sox1, and Vim, which were expressed in all the rDT DPSC isolates except dpsc-3882.

To determine if the microRNA expression was associated with these changes in mRNA expression, several neuronal differentiation-associated microRNAs were screened—including miR-27, miR-125, miR-128, miR-135, miR-140, miR-145, miR-218, and miR-410. Expression of the positive control miR-16 was observed among all the DPSC isolates; however, no expression of miR-125, miR-128, miR-135, miR-140, miR-218, or miR-410 was observed. In addition, the differential expression of miR-27 and miR-145 was found among a subset of the rDT DPSC isolates. More specifically, miR-27 expression was found among dpsc-7089, dpsc-3882, dpsc-9765, and dpsc-3924 but not with dpsc-5653. Moreover, the expression of miR-145 was observed among dpsc-7089, dpsc-3882, and dpsc-965, but not among tdpsc-5653 or dpsc-3924. Finally, the administration of the bFGF and EGF was sufficient to inhibit the expression of miR-27 and miR-145 among all the rDT DPSC isolates.

Analysis of the results of the qPCR screening for the ICST, MSC, and neuronal biomarkers was compiled (Table 4). These data demonstrated that all the DPSC isolates expressed the positive control ICST biomarkers CD90 and CD105, but not the negative control biomarker CD45. Expression of the MSC biomarkers NANOG, Nestin, Sox2, and Oct 4 was observed in all the rDT and iDT DPSC isolates, with the expression of Nestin observed among all the sDT DPSC isolates and NANOG for the dpsc-17322 only. Expression of neuronal differentiation biomarkers was only observed in the post-treatment groups of the rDT DPSC isolates, including beta III tubulin and NFM. Additional biomarkers Pax, Sox1, and Vim were observed in all the rDT group except dpsc-3882, but not among the iDT or sDT DPSC isolates. Finally, expression of miR-27 was observed among all the rDT isolates except the dpsc-5653, while miR-145 was observed only among the dpsc-7089, dpsc-3882, and dpsc-9765. Interestingly, the expression of miR-27 and miR-145 was found among the sDT isolates in the pre-treatment groups, but this expression was lost in the post-treatment groups. No expression of the neuronal differentiation-associated microRNAs miR-125, miR-128, miR-135, miR-140, miR-218, or miR-410 was observed among any of the rDT, iDT, or sDT isolates.

## 4. Discussion

The main objective of this study was to analyze the expression of microRNAs previously identified as regulators of neuronal differentiation among MSCs, such as miR-27, miR-125, miR-128, miR-135, miR-140, miR-145, miR-218, and miR-410, among DPSCs under experimental conditions demonstrated to initiate neural induction pathways [39,40]. The results of this study revealed that differential expression of miR-27 and miR-145 was observed among the stimulus-responsive DPSC isolates. Although miR-145 has been observed in early osteogenic differentiation among the DPSCs, this may be the first observation of miR-27 or miR-145 in any study of neuronal differentiation [44].

Previous studies of development have identified miR-27 as a positive regulator of cartilage differentiation among adipose-derived MSCs [45,46]. In addition, miR-27 has also been identified as highly-enriched in the pharyngeal arches, mainly functioning as a positive regulator of chondrogenesis through complex and dynamic interactions with focal adhesion kinase [47]. Interestingly, miR-27 has also been identified as an early regulator of Hox gene expression related to temporal and spatial differentiation in neuronal differentiation during early spinal cord development [48]. The identification in this current study of miR-27 as one of the differentially expressed microRNAs among the DPSCs that may be down-regulated by the neuronal differentiation stimuli may indicate that this microRNA could be an important negative regulator for neuronal differentiation.

Other studies of developmental biology have identified miR-145 as an important mediator of cartilage differentiation among MSCs—although this regulatory mechanism may be highly dependent upon the co-expression of miR-140 [49,50]. Although this current study found the differential expression of miR-145 among the pre-treatment DPSCs, no expression of miR-140 was observed in any of the DPSC isolates. In addition, some evidence has suggested that miR-140 acts in combination with other important microRNAs to regulate the development, such as the coordinated interactions between miR-140 and miR-143 that modulate smooth muscle cell fate and plasticity [51,52]. Similar mechanisms have also been observed in some studies of MSC neural differentiation, demonstrating miR-145 interactions with miR-29 and miR-124 at specific points during development or differentiation [53,54]. However, miR-145 has also been shown to independently regulate neuronal differentiation through interactions with Sox-2 and was therefore identified as a potential microRNA of interest in a narrative review of potential microRNAs that may function to regulate the DPSCs [55,56]. The observation that the administration of growth factors to induce neuronal differentiation among DPSC may down-regulate the expression of this microRNA, may suggest that this could also be part of an important negative regulatory feedback mechanism.

The current study results also demonstrated that previously identified microRNAs important in neural development and differentiation may not be expressed among the DPSCs under growth-factor-induced conditions. For example, although miR-125 has been shown to function in neural specification among embryonic stem cells, this microRNA may also be temporally expressed to function later in the development as a regulator of terminal neural differentiation and olfactory bulb specification [57,58]. Similarly, miR-128 may regulate neural differentiation in conjunction with miR-124 and miR-137 in highly coordinated networks that modulate more than 900 genes during development and may not therefore be expressed in laboratory settings of DPSCs under experimental settings involving controlled experimental stimuli [59]. Finally, other models of neuronal differentiation among MSCs have used alternative stimuli, such as the fibroblast growth factor 2 (FGF-2), which may activate the overlapping and divergent pathways that may be associated with miR-218 expression—which was not observed in the current study of DPSC under EGF and bFGF administration [60,61].

These differences highlight some of the key limitations that should be considered when evaluating the conclusions that can be drawn from these results. First, the process of long-term cryopreservation and storage may have a deleterious effect on the DPSCs that were previously collected and kept in a biorepository [62,63]. In addition, due to the nature of the study approval and project parameters only DPSCs collected at this institution and stored in the biorepository were available for analysis, which suggests that these results will need to be assessed and validated among other DPSCs to confirm if these observations are reliable and consistent [64,65]. Finally, other studies of MSCs and neuronal differentiation have used other growth factors for induction and stimulation, such as nerve growth factor (NGF) and insulin-like growth factor (IGF), which may stimulate other pathways and may involve differential regulation not observed in the current study, which utilized EGF and bFGF stimulation of DPSCs [66,67,68].

## 5. Conclusions

This study confirmed previous observations of DPSC responsiveness to bFGF and EGF stimulation of DPSCs that were associated with neuronal differentiation. In addition, this study revealed that some microRNAs involved in the neuronal differentiation of MSCs were also differentially expressed among the DPSCs, including miR-27 and miR-145. Finally, this study revealed that the stimulation of these DPSCs with bFGF and EGF down-regulated miR-27 and miR-145 expression in all of the growth factor-responsive DPSCs but not among all of the non-responsive DPSCs, which suggests that further investigation of the downstream targets of these microRNAs may be warranted.

## Figures and Tables

**Table 1:** Sequence of the validated qPCR primers used.

Primers	Sequence
Positive control primers	
Forward primer, Beta actin	5′-GTG GGG TCC TGT GGT GTG-3′
Reverse primer, Beta actin	5′-GAA GGG GAC AGG CAG TGA-3′
Forward primer, GAPDH	5′-ATC TTC CAG GAG CGA GAT CC-3′
Reverse primer, GAPDH	5′-ACC ACT GAC ACG TTG GCA GT-3′
MSC primers	
Forward primer, Nestin	5′-CGT TGG AAC AGA GGT TGG AG-3′
Reverse primer, Nestin	5′-TCC TGA AAG CTG AGG GAA G-3′
Forward primer, NANOG	5′-GCT GAG ATG CCT CAC ACG GAG-3′
Reverse primer, NANOG	5′-TCT GTT TCT TGA CTG GGA CCT TGT C-3′
Forward primer, Oct4	5′-TGG AGA AGG AGA AGC TGG AGC AAA A-3′
Reverse primer, Oct4	5′-GGC AGA TGG TCG TTT GGC TGA ATA-3′
Forward primer, Sox2	5′-ATG GGC TCT GTG GTC AAG TC-3′
Reverse primer, Sox2	5′-CCC TCC CAA TTC CCT TGT AT-3′
ISCT control primers	
Forward primer, CD45	5′-CAT ATT TAT TTT GTC CTT CTC CCA-3′
Reverse primer, CD45	5′-GAA AGT TTC CAC GAA CGG-3′
Forward primer, CD90	5′-ATG AAC CTG GCC ATC AGC A-3′
Reverse primer, CD90	5′-GTG TGC TCA GGC ACC CC-3′
Forward primer, CD105	5′-CCA CTA GCC AGG TCT CGA AG-3′
Reverse primer, CD105	5′-GAT GCA GGA AGA CAC TGC TG-3′
Neuronal differentiation primers	
Forward primer, BIII tubulin	5′-GGC CAA GGG TCA CTA CAC G-3′
Reverse primer, BIII tubulin	5′-GCA GTC GCA GTT TTC ACA CTC-3′
Forward primer, NFM	5′-GCT CGT CAT TTG CGC GAA TAC-3′
Reverse primer, NFM	5′-TTT CTG TAC GCA GCG ATT TCT AT-3′
Forward primer, Pax6	5′-TGG GCA GGT ATT ACG AGA CTG-3′
Reverse primer, Pax6	5′-ACT CCC GCT TAT ACT GGG CTA-3′
Forward primer, Sox1	5′-CAG TAC AGC CCC ATC TCC AAC-3′
Reverse primer, Sox1	5′-GCG GGC AAG TAC ATG CTG A-3′
Forward primer, Vim	5′-GAC GCC ATC AAC ACC GAG TT-3′
Reverse primer, Vim	5′-CTT TGT CGT TGG TTA GCT GGT-3′
microRNA primers	
Forward primer, miR-16	5′-TAG CAG CAC GTA AAT ATT GGC G-3′
Reverse primer, miR-16	5′-TGC GTG TCG TGG AGT C-3′
Forward primer, miR-27	5′-ATA TGA GAA AAG AGC TTC CCT GTG-3′
Reverse primer, miR-27	5′-CAA GGC CAG AGG AGG TGA G-’3′
Forward primer, miR-125	5′-GCC CTC CCT GAG ACC TCA A-3′
Reverse primer, miR-125	5′-GTG CAG GGT CCG AGG T-3′
Forward primer, miR-128	5′-TCT CCT AAA GAG CCC GAA CA-3′
Reverse primer, miR-128	5′-TTG CAT TCA TAG CTG CAT CC-3′
Forward primer, miR-135	5′-CGA TAT GGC TTT TTA TTC CTA -3′
Reverse primer, miR-135	5′-GAG CAG GGT CCG AGG T -3′
Forward primer, miR-140	5′-GGG CAG TGG TTT TAC CCT A -3′
Reverse primer, miR-140	5′-CAG TGC GTG TCG TGG AGT -3′
Forward primer, miR-145	5′-AGA GAA CTC CAG CTG-3′
Reverse primer, miR-145	5′-GGC AAC TGT GGG GTG-3′
Forward primer, miR-218	5′-TCG GGC TTG TGC TTG ATC T-3′
Reverse primer, miR-218	5′-GTG CAG GGT CCG AGT G-3′
Forward primer, miR-410	5′-CCG CAC GAT ATA ACA CAG ATG-3′
Reverse primer, miR-410	5′-GTG CAG GGT CCG AGG TAT TC-3′

**Table 2:** Baseline characteristics of the DPSC isolates.

	Rapid Doubling Time (rDT)	Intermediate Doubling Time (iDT)	Slow Doubling Time (sDT)	Viability (Baseline)
dpsc-7089	1.9 days			62%
dpsc-3882	1.8 days			58%
dpsc-5653	1.9 days			77%
dpsc-9765	2.1 days			69%
dpsc-3924	2.2 days			66%
**rDT average**	**1.98 days**			**66.40%**
dpsc-8604		5.1 days		50%
dpsc-8124		5.4 days		53%
dpsc-9894		5.2 days		66%
**iDT average**		**5.23 days**		**56.30%**
dpsc-11418			10.2 days	63%
dpsc-17322			10.6 days	56%
dpsc-11836			12.1 days	57%
dpsc-11750			11.9 days	62%
**sDT average**			**11.2 days**	**59.50%**

**Table 3:** RNA isolation and cDNA synthesis.

	RNA Concentration	RNA Purity (A260:A280 Ratio)	cDNA Concentration	DNA Purity (A260:A280 Ratio)
dpsc-7089	481 ng/µL	1.88	1541 ng/µL	1.89
dpsc-3882	357 ng/µL	1.67	1480 ng/µL	1.92
dpsc-5653	443 ng/µL	1.82	1606 ng/µL	1.82
dpsc-9765	432 ng/µL	1.73	1587 ng/µL	1.9
dpsc-3924	320 ng/µL	1.82	1527 ng/µL	1.92
**rDT average**	**406.6 ng/µL**	**1.78**	**1548.2 ng/µL**	**1.89**
dpsc-8604	447 ng/µL	1.84	1589 ng/µL	1.8
dpsc-8124	302 ng/µL	1.66	1551 ng/µL	1.89
dpsc-9894	577 ng/µL	1.85	1561 ng/µL	1.81
**iDT average**	**442.0 ng/µL**	**1.78**	**1567.0 ng/µL**	**1.83**
dpsc-11418	484 ng/µL	1.77	1607 ng/µL	1.81
dpsc-17322	519 ng/µL	1.78	1610 ng/µL	1.89
dpsc-11836	321 ng/µL	1.86	1489 ng/µL	1.86
dpsc-11750	567 ng/µL	1.85	1492 ng/µL	1.92
**sDT average**	**472.8 ng/µL**	**1.81**	**1549.5 ng/µL**	**1.87**

**Table 4:** qPCR analysis of RNA expression from DPSC isolates.

	ICST Biomarkers	MSC Biomarkers	Neuronal Biomarkers (Post-Treated)	microRNA Expression
rDT dpsc-7089 dpsc-2883 dpsc-5653 dpsc-9765 dpsc-3924	CD90, CD105 (all rDT) CD45 (no rDT)	NANOG Nestin Oct4 Sox2 (all rDT)	BIII tubulin, NFM (all rDT) Pax, Sox1, Vim (all rDT but dpsc-3882)	miR-27 (all rDT but dpsc-5653) miR-145 (dpsc-7089, dpsc-3882, dpsc-9765) miR-125, miR-128 miR-135 miR-140 miR-218 miR-410 (no rDT)
iDT dpsc-8604 dpsc-8124 dpsc-9894	CD90 (all iDT) CD105 (all iDT) CD45 (no iDT)	NANOG Nestin Oct4 Sox2 (all iDT)	BIII tubulin NFM Pax Sox1 Vim (no iDT)	miR-27 miR-125, miR-128 miR-135 miR-140 miR-145 miR-218 miR-410 (no iDT)
sDT dpsc-11418 dpsc-17322 dpsc-11836 dpsc-11750	CD90 (all sDT) CD105 (all sDT) CD45 (no sDT)	NANOG (dpsc-17322) Nestin (all sDT) Sox2, Oct4 (no sDT)	BIII tubulin NFM Pax Sox1 Vim (no sDT)	miR-27 (all sDT) miR-145 (all sDT) miR-125, miR-128 miR-135 miR-140 miR-218 miR-410 (no sDT)

## Data Availability

Due to the study protocol parameters and protocol specifications, all requests for study data must be directed to the study authors.
